# Functional results and photic phenomena with new extended-depth-of-focus intraocular Lens

**DOI:** 10.1186/s12886-019-1201-3

**Published:** 2019-08-28

**Authors:** Bert C. Giers, Ramin Khoramnia, Dorottya Varadi, Hannah Wallek, Hyeck-Soo Son, Mary S. Attia, Gerd U. Auffarth

**Affiliations:** The David J Apple Center for Vision Research, Department of Ophthalmology, University of Heidelberg, Im Neuenheimer Feld 400, 69120 Heidelberg, Germany

**Keywords:** Cataract, Intraocular Lens, Multifocal, Visual outcomes

## Abstract

**Background:**

Evaluation of clinical and functional results of a new extended depth of focus intraocular lens (EDOF-IOL).

**Methods:**

Fourteen cataract patients (28 bilateral implantations) were assessed for uncorrected (UDVA) and corrected (CDVA) distance visual acuities; uncorrected (UNVA), distance-corrected (DCNVA) and best corrected (CNVA) near visual acuities; and uncorrected (UIVA) and distance-corrected (DCIVA) intermediate visual acuities - as well as binocular defocus curves. Photopic and mesopic contrast sensitivity was recorded. Reading acuity was evaluated using an electronic reading desk at fixed distances and at the patient’s preferred near and intermediate distances. Visual symptoms were assessed with a halo and glare simulator plus a patient questionnaire which also recorded quality of life.

**Results:**

Median postoperative monocular UDVA was 0.13logMAR (range − 0.08 to 0.42logMAR), median CDVA was − 0.01logMAR (range − 0.20 to 0.22logMAR), median UIVA at 80 cm was − 0.05logMAR (range − 0.18 to 0.58logMAR) and median UNVA at 40 cm was 0.14logMAR (range − 0.10 to 0.64logMAR). Binocular uncorrected reading acuity was 0.10logMAR at 40 cm and 0.11logMAR at 80 cm. Patients preferred a median intermediate reading distance of 62.8 cm over the predetermined 80 cm, which allowed them to read smaller letter size but did not improve reading acuity. Patients reported a high rate of spectacle independence and satisfaction in everyday life and little to no dysphotopsia.

**Conclusion:**

The Mini WELL Ready IOL provided good postoperative functional results at far and intermediate distances and improved the visual and reading acuity at reading distance. The lens caused little to no dysphotopsia.

**Trial registration:**

The study protocol was registered at the German Clinical Trials Register: DRKS00007837 (Registered Date: March 9th, 2015).

## Background

In recent years, multifocal intraocular lenses (MIOLs) were developed for patients who do not want to wear spectacles when using a desktop computer or mobile phone. The lenses either have a low to moderate addition for intermediate distances or are trifocal with an additional intermediate focus [1–3].

Trifocal IOLs deliver good functional vision at different focal planes to achieve spectacle independence [4–8]. A new development is extended-depth-of-focus IOLs (EDOF-IOLs), that aim to provide a continuous range of vision instead of multiple distinct foci. The first EDOF-IOL was the Tecnis Symfony (Abbott Medical Optics, Abbott Park, NY, USA), combining an apodized-diffractive surface with an echelette-design. Initial clinical results appear promising [9–13].

A considerable disadvantage of diffractive designs is loss in contrast sensitivity due to reduced effective light energy reaching each focal plane. Perception of halos and glare, is another side-effect: arising from superposition of multiple images on the retina [14, 15]. Patient dissatisfaction can occur despite the functional visual results achieved.

An optical design not primarily based on diffraction ought to minimize these disadvantages. We examined the clinical outcomes with the Mini WELL Ready (SIFI, Catania, Italy), a new EDOF-IOL that relies on spherical aberrations of opposite signs to create an elongated focus. Standard visual acuity was tested using charts for predefined distances and an electronic reading desk was used for visual acuity assessment at individually chosen distances [16, 17]. Furthermore, contrast sensitivity as well as subjective perception of glare and halos were assessed to further characterize the clinical performance.

## Methods

### Patients and procedure

This prospective, non-randomized clinical study was approved by the local Ethics Committee and performed in accordance with the tenets of the Declaration of Helsinki. Patients were recruited between August 2016 and May 2017 for bilateral implantation of the Mini WELL Ready IOL. All patients gave informed consent form prior to inclusion to the study. Otherwise, they were not involved in the design of the study. Patients were aged 18 years or older, had a postoperative expected uncorrected visual acuity of 0.2logMAR (20/32) or better as well as a postoperative expected corneal astigmatism under 1.0D (diopters). Patients were excluded with previous ocular surgery or other ocular and monocular pathologies, except cataract, that could affect postoperative visual acuity.

### The IOL

**Fig. 1:**
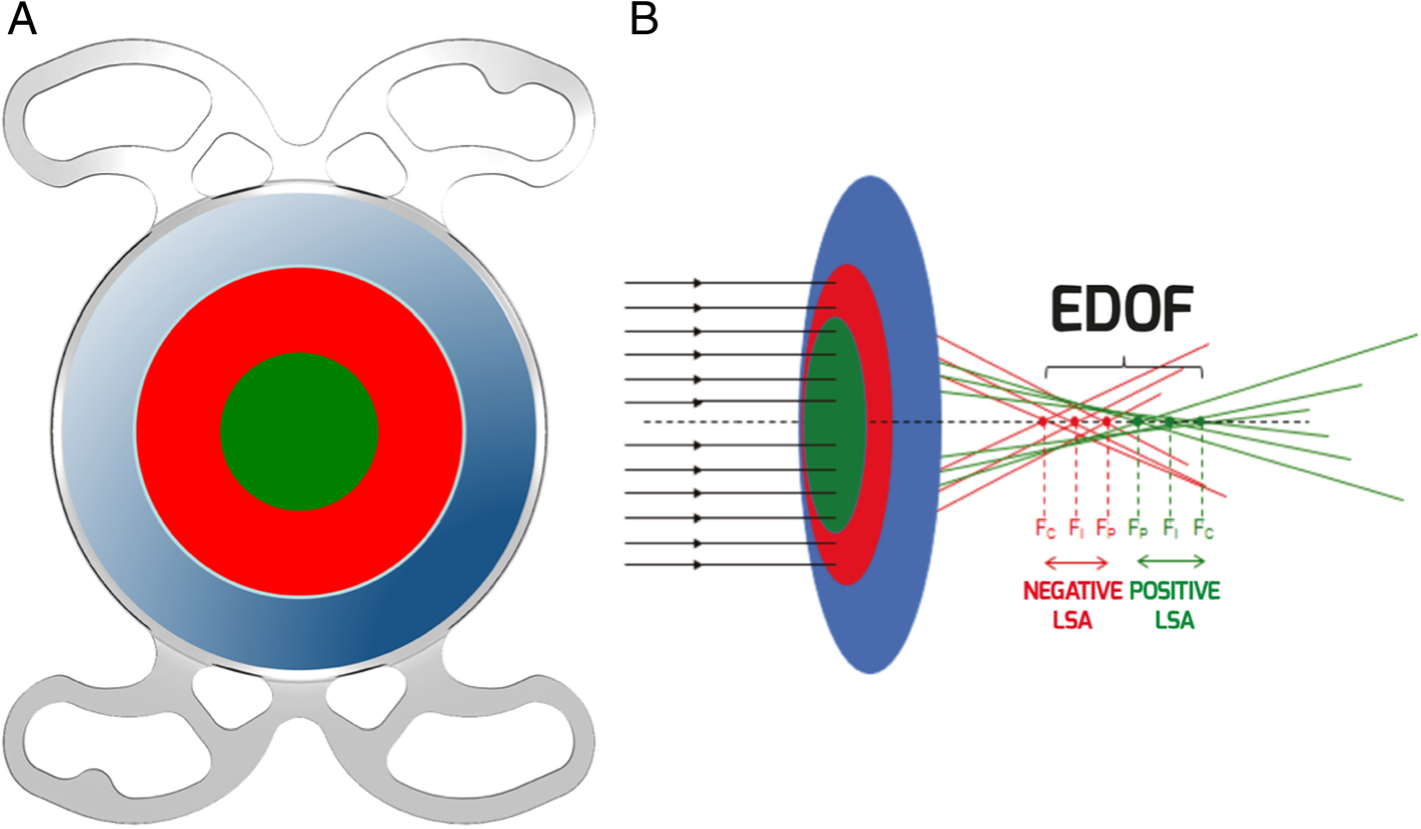
Schematic illustration of the Mini WELL Ready. **a**) D1 is shown in green, D2 in red, and D3 in blue. **b**) In D1, the central rays (Fc, green) come into focus behind the peripheral rays (Fp, green), while in D2, the central rays (Fc, red) come into focus in front of peripheral rays (Fp, red). LSA = Longitudinal spherical aberration; EDOF = Extended-depth-of-focus

The SIFI Mini WELL Ready is a preloaded, single-piece hydrophilic acrylic IOL with a hydrophobic surface. The overall diameter is 10.75 mm with four closed-loop haptics with 5-degree angulation. The biconvex optic of 6 mm diameter has three annuli, an outer monofocal zone and two inner zones with spherical aberrations of opposite signs (Fig. 1). The innermost zone, or D1, is 1.8 mm wide and has a positive spherical aberration, creating the intermediate focus. The middle zone, or D2, is 3.0 mm wide and has a negative spherical aberration, contributing to near focus. The outermost zone, or D3, is a monofocal optic with a diameter of 6.0 mm that is responsible for creating the far focus. The lens features an equivalent addition of + 3.0D corresponding to a spectacle plane addition of + 2.4D. Power ranges from 0 to +30D (0.5D increments from + 10.5 to 30.0D). The company’s estimated A-constant is 118.6.

### Surgical procedure

The same experienced surgeon (G.U.A.) performed all surgeries using topical or general anesthesia. A 12 o’clock clear corneal incision preceded manual curvilinear (*n* = 13) or a femtosecond laser-assisted capsulorhexis (*n* = 1), followed by standard phacoemulsification. For femtosecond laser-assisted capsulorhexis, the size of the rhexis was 5.0 mm. For manual curvilinear capsulorhexis, the intended size of the rhexis was also 5.0 mm. The IOL was implanted in the capsule. Postoperative topical medication was a combination of antibiotic and a steroid for 2 weeks. IOL power for targeted emmetropia was calculated with the Holladay formula; except where axial length was less than 22 mm or over 25 mm, (as measured on the IOLMaster (Carl Zeiss Meditec)), the Haigis formula was used.

### Postoperative examinations

Two to 4 months after surgery, patients were evaluated for visual acuity, reading performance, contrast sensitivity, photic phenomena and patient satisfaction. In addition to these monocular examinations, “real-life” binocular performance was evaluated.

### Visual acuity and Reading acuity examinations

Uncorrected and distance-corrected visual acuities were determined using Early Treatment Diabetic Retinopathy Study (ETDRS) charts (Precision Vision Inc., USA). The 4 m charts were used for distance vision, the 80 cm charts for intermediate vision, and the 40 cm charts for near vision. For the binocular defocus curve, patients were tested distance-corrected using a 4 m ETDRS chart and adding + 5.0 to − 3.0D in 0.5D increments. Standardized illumination of 500 lx was used, according to the DIN EN 12464–1 norm.

### Electronic Reading desk

Reading performance was evaluated with the Salzburg Reading Desk Version RDFD 1.0 (SRD Vision LLC, USA) at 40 cm fixed distance for near and 80 cm for intermediate visual acuity as well as at the patient’s preferred near and intermediate distances [18, 19]. Patients read logarithmically-scaled Colenbrander sentences while reading distance is measured continuously using video-stereophotogrammetry. Overall reading acuity is calculated automatically in logMAR, with consideration of the reading distance in centimeters and the log-scaled print size of the smallest readable sentence with a minimum velocity of 80wpm.

### Patient questionnaire

Patients responded to questions on visual perception and satisfaction in performing daily activities, rating the occurrence of visual problems from 0 to 10, where 0 is complete absence of a problem and 10 is strong discomfort.

### Contrast sensitivity and photic phenomena

Contrast sensitivity was measured using a stereo optical functional acuity contrast test (F.A.C.T., CSV-1000, VectorVision, Greenville, OH) adjusted for distance under photopic (85 cd/m^2^) and mesopic (3 cd/m^2^) conditions.

To assess photic phenomena, we used a PC-based simulator software (Halo & Glare Simulator, Eyeland-Design Network GmbH, Vreden, Germany) where patients select from different kinds of preset halos and glare and then separately adjust for size and intensity on a slide bar with simultaneous visual representation on the screen. What the patient perceives is classified into three types: diffuse halo, starburst or a single distinct halo ring. Patients are asked to adjust the slide bars so that the image on the screen gives a representation of how they perceive photic phenomena around light sources during nighttime driving. The slide bar positions for each item are translated into numeric values between 0 (minimum) and 100 (maximum) on a visual analogous scale.

### Statistical analysis

Using SPSS version 21 for Windows, (IBM, Armonk, NY), the results from binocular examinations were analyzed using Wilcoxon rank-sum test for paired non-parametric data. For monocular examinations, the linear mixed model of SPSS software was used. For all tests, the same level of significance was adopted (*p* < 0.05). The questionnaires and halometry results were analyzed descriptively.

## Results

### Visual acuity and refraction

Two patients were excluded from analysis: one had developed bilateral postoperative macular edema and another had died from heart-failure. Thus, only 28 eyes of 14 patients were analyzed, nine men (64.3%) and five women (35.7%). Median age was 66 years (range: 52 to 82 years).

The median spherical IOL power was 20D (range 14 to 24D). The median preoperative spherical equivalent was 0.00 D (range − 2.50 to + 2.38 D), target spherical equivalent was − 0.23 D (range − 0.56 to + 0.25 D) and median achieved postoperative spherical equivalent was 0.00 D (range − 1.63 to + 2.13 D). Median postoperative residual cylinder was − 0.50 D. There was no statistically significant difference between achieved and target refraction (*p* = 0.24). Fourteen eyes (50%) were within ±0.50 D and 24 (85.7%) were within ±1.00 D of the intended value.

Postoperative median UDVA (monocular) was 0.13logMAR (range − 0.08 to 0.42logMAR), median UIVA at 80 cm was − 0.05logMAR (range − 0.18 to 0.58logMAR) and median UNVA at 40 cm was 0.14 log MAR (range − 0.10 to 0.64logMAR). The median UDVA (binocular) was 0.05logMAR (range − 0.08 to 0.22logMAR). The median CDVA improved significantly (*p* < 0.001) from 0.43logMAR preoperatively (range hand movements to 0.04logMAR) to − 0.01logMAR postoperatively (range − 0.20 to 0.22logMAR). Postoperative median DCIVA was 0.03logMAR (range − 0.02 to 0.58logMAR) and median CNVA (median add. + 1.50 D, range 0 to + 2.75 D) was − 0.08 log MAR (range − 0.20 to 0.34 log MAR). 79% of tested eyes achieved both a UDVA and a UNVA of 0.20logMAR (20/32 Snellen or 0.63 Decimal) or better and 89% of eyes achieved a UIVA of 0.20logMAR or better (Table 1).

**Table 1:** Monocular (*n* = 28 eyes of 14 patients) visual acuity results in logMAR

	Mean	SD	Median	Min.	Max.
UDVA	0.13	0.13	0.13	−0.08	0.42
CDVA	0.00	0.11	−0.01	−0.20	0.22
UIVA	0.01	0.17	−0.05	−0.18	0.58
DCIVA	0.04	0.18	0.03	−0.02	0.58
UNVA	0.17	0.20	0.14	−0.10	0.64
DCNVA	0.20	0.21	0.18	−0.10	0.62
CNVA	−0.03	0.14	−0.08	−0.20	0.34
Near Add. (D)	+ 1.59	0.72	+ 1.50	0.00	+ 2.75

*UDVA* = uncorrected distance visual acuity, *CDVA* = corrected distance visual acuity, *UIVA* = uncorrected intermediate visual acuity, *DCIVA* = distance-corrected intermediate visual acuity, *UNVA* = uncorrected near visual acuity, *DCNVA* = distance-corrected near visual acuity, *CNVA* = corrected visual acuity, *Near Add*. = near addition (in diopters of spherical power)

**Fig. 2:**
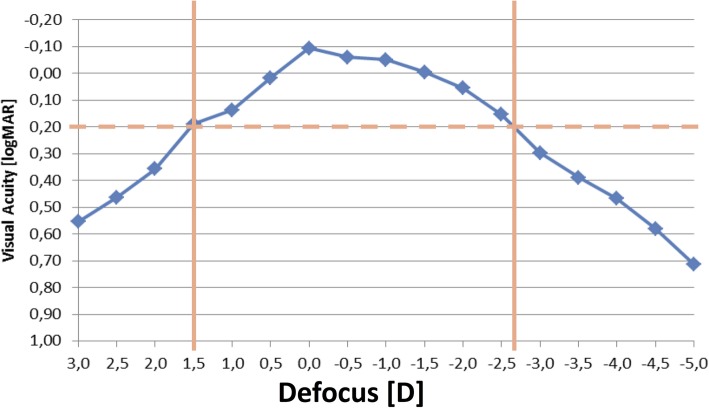
Binocular distance-corrected defocus curve (*n* = 14 patients)

The binocular defocus curve shows a broad plateau with a visual acuity of 0.20logMAR or better between + 1.50 and − 2.50D (Fig. 2).

### Reading performance

Table 2 summarizes the Salzburg Reading Desk results of binocular uncorrected and distance-corrected reading acuity at predetermined near (40 cm) and intermediate (80 cm) distances as well as at the subjectively preferred reading distance for both near and intermediate distances. The reading performance could only be tested in 11 subjects as three subjects could not speak any language that is supported by the Salzburg Reading Device fluently. There was no statistically significant difference between the predetermined near distance of 40 cm (median 40.3 cm) and the individually preferred reading distance (median 39.5 cm) both for uncorrected (*p* = 0.21) and distance-corrected (*p* = 0.92) testing and no significant changes were observed for reading acuity, letter size and reading speed. Patients preferred an intermediate reading distance of approximately 60–65 cm (median 62.8 cm and 60 cm for uncorrected and distance-corrected binocular testing respectively) over the predetermined, 80 cm intermediate distance (*p* = 0.003), which allowed them to read smaller letter sizes (*p* = 0.004 and 0.045 for uncorrected and distance-corrected testing respectively). However, the reading acuity results did not significantly differ between the predetermined and the patient’s preferred intermediate distance.

**Table 2:** Median binocular (*n* = 11 patients) uncorrected and distance-corrected reading performance for near and intermediate distances with the Salzburg Reading Desk. wpm = words per minute

	Near Distance (40 cm)	Preferred Near Distance	Intermediate Distance (80 cm)	Preferred Intermediate Distance
Uncorrected				
Visual Acuity (logMAR)	0.10	0.13	0.11	0.11
Distance (cm)	40.30	39.50	79.20	62.80
Letter Size (log-scaled)	1.25	1.25	1.25	1.00
Reading Speed (wpm)	92.0	92.0	110.0	100.0
Distance-Corrected				
Visual Acuity (logMAR)	0.21	0.19	0.10	0.12
Distance (cm)	40.30	38.90	79.40	60.00
Letter Size (log-scaled)	1.50	1.50	1.25	1.00
Reading Speed (wpm)	97.0	102.0	103.0	97.0

### Photic phenomena

**Fig. 3:**
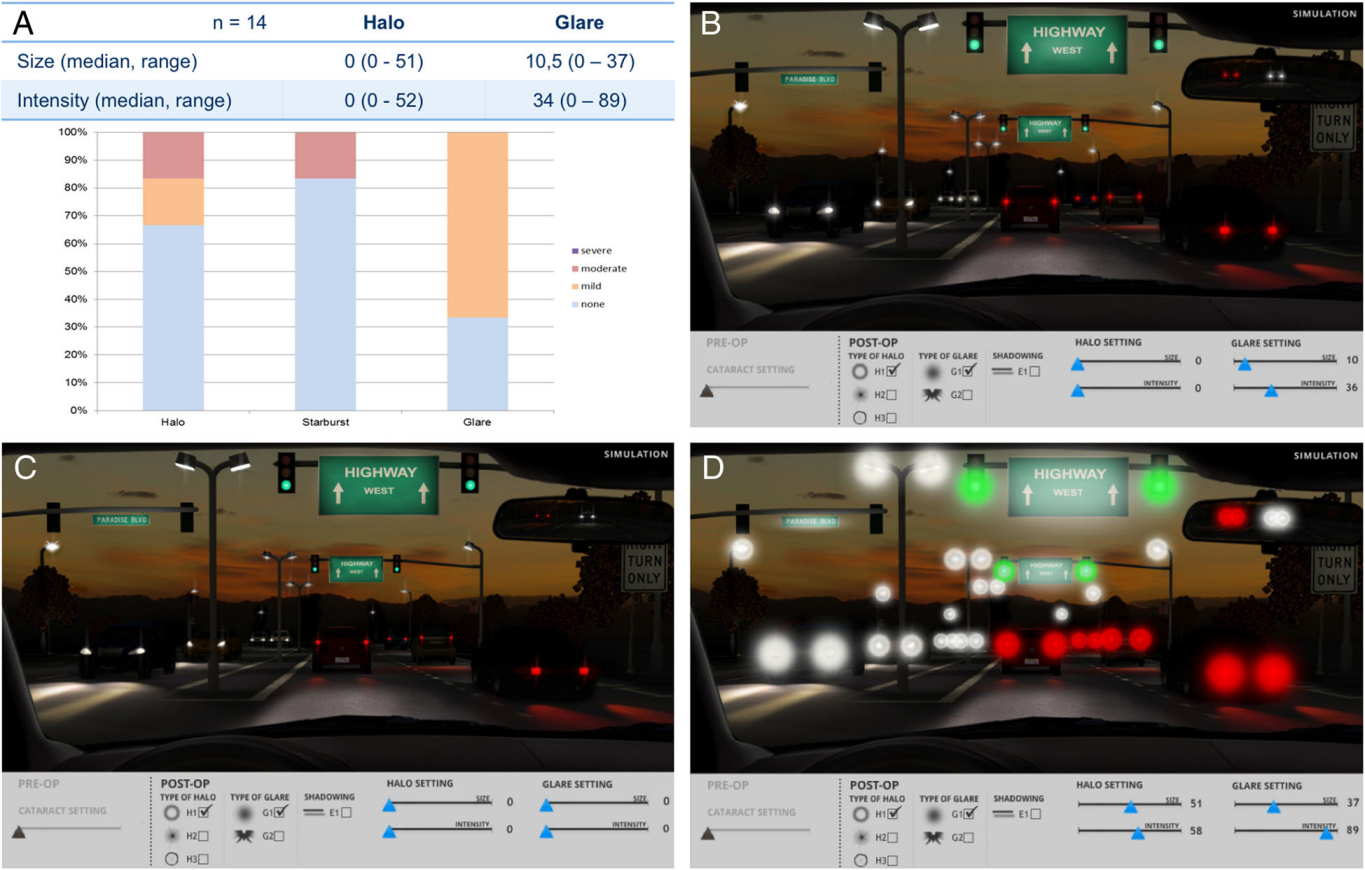
Halometry. (**a**) Results of Halos and Glare Simulator (Median, Range; Visual Analogous Scale) as well as quantity and severity of photic phenomena. (**b**) Mean values for perceived Halos and Glare. (**c**) Minimum values and (**d**) maximum values for perceived Halos and Glare. No patient perceived photic phenomena of comparable severity, since the simulation shows the combination of maximum reported values for each individual category (Halo Size, Halo Intensity, Glare Size and Glare Intensity)

**Fig. 4:**
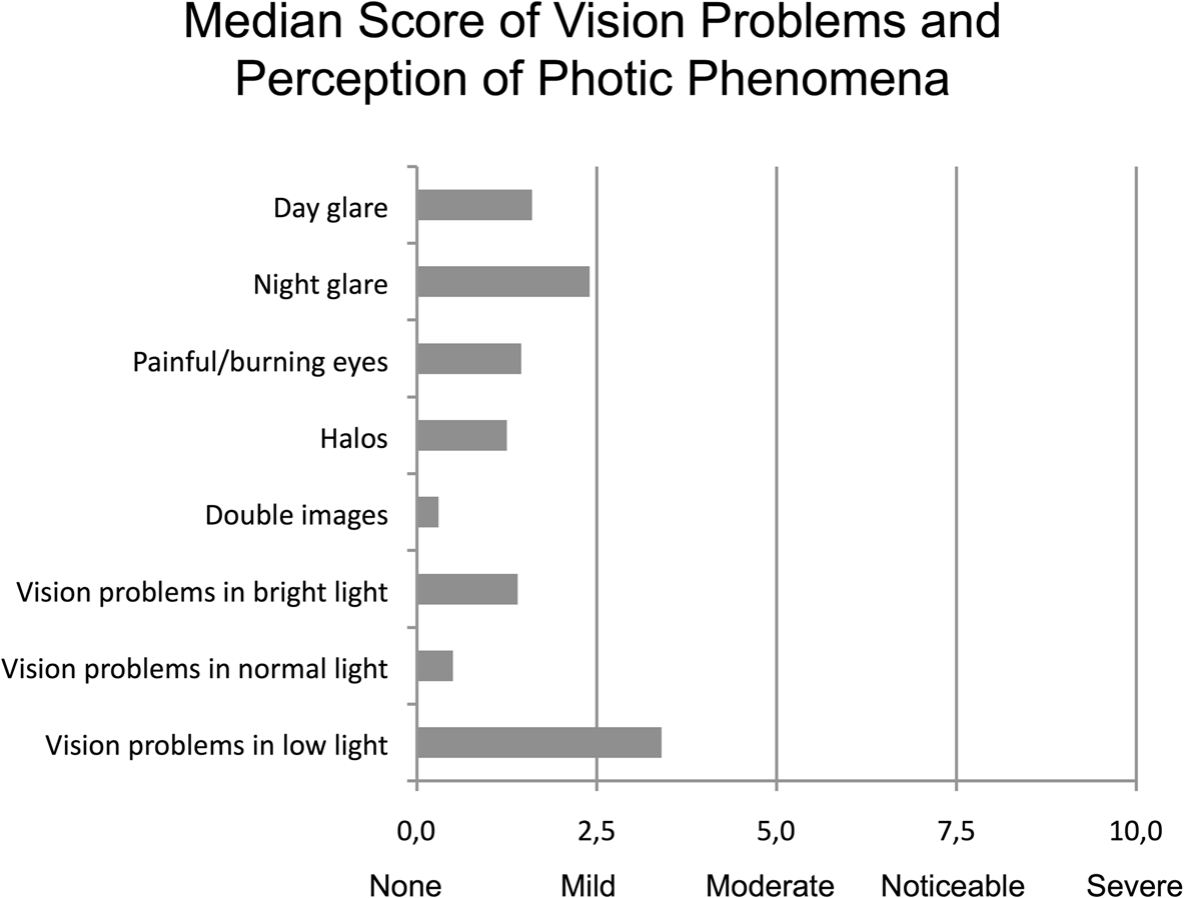
Questionnaire results for vision problems and perception of photic phenomena

54% of patients reported not perceiving any type of halo and 30% reported no glare. 23% saw starburst type halos. The remaining 23% reported a classical concentric halo around light sources. Median scores for halo size and intensity were 0 (visual analogous scale) median scores for glare size and intensity were 10 and 36 (Fig. 3). These results correspond to the questionnaire responses, where respondents were less than mildly or not at all bothered by day glare, night glare and perception of halos (median scores 1.6, 2.4 and 1.3 respectively; Fig. 4).

### Patient satisfaction

**Fig. 5:**
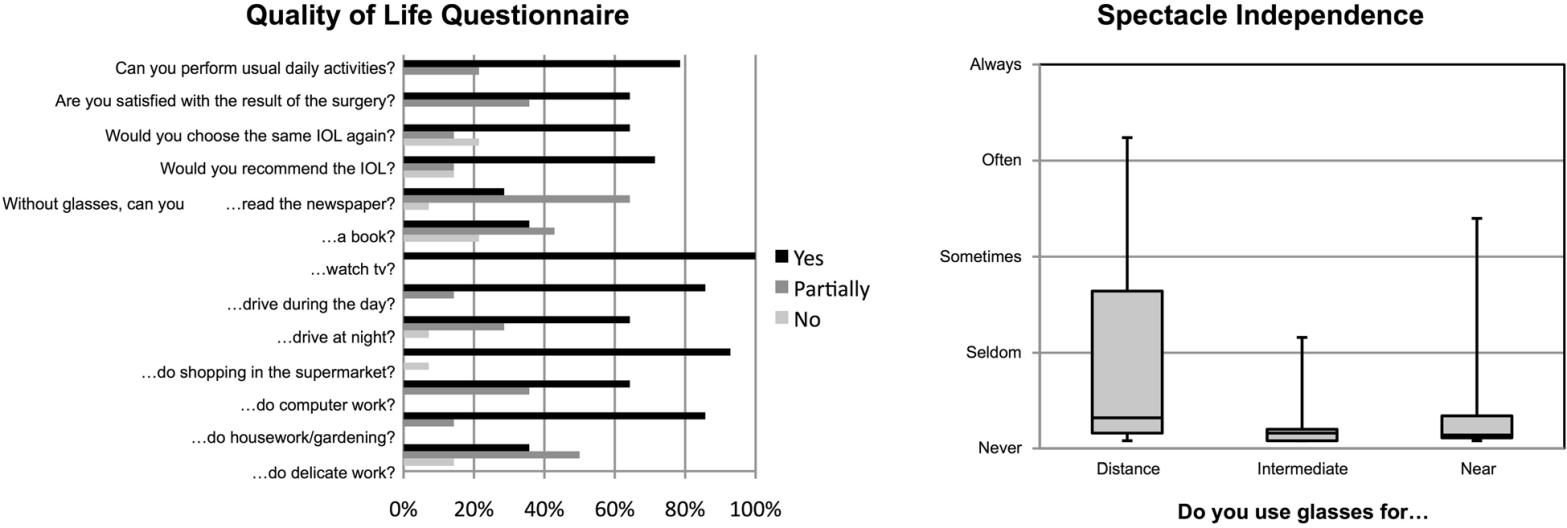
Quality of Life Questionnaire and Spectacle Independence

All 14 patients completed the questionnaire. 78.6% stated they were able to perform their usual daily activities without additional spectacle correction. 71.4% would recommend the IOL to family and friends. Whereas the majority appeared to have little problems with unaided distance vision (watching TV: 100%, driving at day: 85.7%, shopping 92.9%) only 28.6% stated that they were able to read the newspaper and 35.7% were able to read books without glasses (Fig. 5).

The most commonly cited complaint was difficulty in low lighting (median score 3.4 out of 10) followed by night glare (median score 2.4 out of 10). For all other given items, the median score was 1.6 or lower (Fig. 4).

Overall spectacle independence was high for all distances, but with large ranges for distance and near vision (Fig. 5).

## Discussion

The Mini WELL Ready EDOF IOL showed good visual acuity outcomes that are consistent with that of IOLs such as the Tecnis Symfony.

Comparison of the two lenses is not easy because there are wide differences in the examinations performed in published reports on Symfony. Pedrotti et al. describe mean monocular UDVA, UIVA (60 cm) and UNVA values of 0.08 ± 0.12logMAR, 0.24 ± 0.11logMAR and 0.27 ± 0.11logMAR (50 eyes of 25 patients, 3 month follow-up) [12]. Kaymak et al. found a mean monocular UDVA, UIVA (70 cm) and UNVA of 0.02 ± 0.09logMAR, − 0.05 ± 0.08logMAR and 0.33 ± 0.12logMAR (36 eyes of 18 patients, 3 month follow-up) [11]. From other studies, only binocular visual acuity values are available: Ruiz-Mesa et al. report mean binocular UDVA, UIVA and UNVA values of 0.01 ± 0.02logMAR, 0.09 ± 0.08logMAR and 0.17 ± 0.06logMAR for this IOL (20 patients, 1 year follow-up) [13]. Similarly, Cochener et al. found a mean binocular UDVA, UIVA and UNVA of 0.03 ± 0.09logMAR, 0.13 ± 0.16logMAR and 0.21 ± 0.16logMAR (299 patients in the non-monovision group, 4–6 month follow-up) [9].

Our results are generally comparable to those of the aforementioned studies. Median UDVA was 0.13logMAR, perhaps attributable to residual refractive error and a non-optimized A-constant since it was fully-correctable by spectacles (median CDVA − 0.01logMAR). The Mini-Well performed even slightly better at intermediate and near distances with a median UIVA (80 cm) and UNVA of − 0.05 and 0.14logMAR respectively. Similar results were also observed in a multicenter study by Savini et al., who reported an enhanced depth of vision through 2.0 D of defocus, with the best performance at 1.0 and 1.5 D [20].

Our findings also appear to conform with laboratory observations. On the optical bench, Gatinel et al. showed the Symfony behaves rather like a bifocal IOL with a low add power [10], whereas Domínguez-Vicent found the Mini WELL has a broad depth of focus in the intermediate to near vergence [21]. This is also revealed by the defocus curve of the Mini WELL that shows a broad plateau of functional vision overlapping more than 4 diopters of defocus.

For patients seeking spectacle independence in most of everyday activities, the alternative to an EDOF-IOL is a trifocal IOL. All the latest trifocal IOLs use diffractive optics to generate three distinct foci for far, intermediate and near vision. For the AcrySof Panoptix IOL (Alcon Laboratories, Ft. Worth, TX, USA) Lawless et al. report initial results for monocular UDVA, UIVA (60 cm) and UNVA of 0.01 ± 0.10logMAR, 0.30 ± 0.14logMAR and 0.18 ± 0.10logMAR (66 eyes of 33 patients, 6 weeks follow-up) [7] (Table 3). In a comparison of two trifocal IOL designs in 10,084 eyes of 5042 patients, Bilbao-Calabuig et al. reported monocular UDVA, UIVA (80 cm) and UNVA (40 cm) outcomes of 0.04 ± 0.08logMAR, 0.00 ± 0.17logMAR and 0.07 ± 0.10logMAR for the AT Lisa tri 839MP (Carl Zeiss AG, Jena, Germany) and 0.06 ± 0.08logMAR, − 0.01 ± 0.15logMAR and 0.08 ± 0.10logMAR for the FineVision Micro F IOL (PhysIOL, Liege, Belgium) respectively (3 month follow-up) [4]. Other studies showed similar results for these two IOLs [6, 23, 25]. However, it should not be overlooked, that initial studies with the same IOL models in smaller patient cohorts showed different results: Sheppard et al. reported a monocular UDVA of 0.19 ± 0.09 for the FineVision IOL (30 eyes of 15 patients, 2 month follow-up) [22] and Alió et al. found monocular UDVA, UIVA (80 cm) and UNVA (40 cm) values of 0.18 ± 0.13logMAR, 0.20 ± 0.11logMAR and 0.26 ± 0.15logMAR for the same IOL model (40 eyes of 20 patients, 6 month follow-up) [5]. Similarly, for the AT Lisa trifocal IOL Mojzis et al. recorded UDVA, UIVA (66 cm) and UNVA (33 cm) results of − 0.03 ± 0.09logMAR, 0.08 ± 0.10logMAR and 0.20 ± 0.12logMAR (60 eyes of 30 patients, 6 months follow-up) [24], Kretz et al. report a monocular UDVA, UIVA (80 cm) and UNVA (40 cm) of 0.10logMAR, 0.15logMAR and 0.10logMAR (76 eyes of 38 patients, 3 months follow-up) [26] and Mendicute et al. report a binocular UDVA, UIVA (80 cm) and UNVA (40 cm) of 0.03 ± 0.09logMAR, 0.10 ± 0.15logMAR and 0.15 ± 0.14logMAR (104 patients, 3 months follow-up) [8].

**Table 3:** Mean postoperative uncorrected binocular visual acuity results (logMAR) of previous studies on different trifocal intraocular lens models

First Author	IOL Studied	Patients included	Mean postoperative uncorrected binocular visual acuity (logMAR)		
			UDVA	UIVA (*distance*)	UNVA (*distance*)
Lawless et al. [7]	*PanOptix*	33 patients	0.01 ± 0.10	0.30 ± 0.14 (*60 cm*)	0.18 ± 0.10 (40 cm)
Bilbao-Calabuig et al. [4]	*AT Lisa tri 839MP*	2141 patients	0.04 ± 0.08	0.00 ± 0.17 (*80 cm*)	0.07 ± 0.10 (*40 cm*)
Bilbao-Calabuig et al. [4]	*Micro F*	2901 patients	0.06 ± 0.08	−0.01 ± 0.15 (8*0 cm*)	0.08 ± 0.10 (*40 cm*)
Sheppard et al. [22, 23]	*FineVision*	15 patients	0.19 ± 0.09	–	–
Alio et al. [5]	*FineVision*	20 patients	0.18 ± 0.13	0.20 ± 0.11 (*80 cm*)	0.26 ± 0.15 (*40 cm*)
Mojzis et al. [24, 22]	*AT Lisa tri 839MP*	30 patients	−0.03 ± 0.09	0.08 ± 0.10 (*66 cm*)	0.20 ± 0.12 (*33 cm*)
Mendicute et al. [8]	*AT Lisa tri 839MP*	104 patients	0.03 ± 0.09	0.10 ± 0.15 (*80 cm*)	0.15 ± 0.14 (*40 cm*)
Cochener et al. [6]	*FineVision*	99 patients	0.01 ± 0.06	0.08 ± 0.10 (*60 cm*)	0.00 ± 0.04 (*30 cm*)
Kretz et al. [25, 21]	*AT Lisa tri 839MP*	50 patients	0.06 ± 0.10	0.09 ± 0.10 (*66 cm*)	0.06 ± 0.05 (*40 cm*)
Jonker SM et al. [23, 25]	Micro F	15 patients	0.01 ± 0.11	0.32 ± 0.15 (*70 cm*)	0.15 ± 0.13 (*40 cm*)

Few studies have explicitly examined reading performance after MIOL implantation [18, 19, 27–29]. The Salzburg Reading Desk measures reading acuity under close-to real-life conditions with simultaneous and continuous measurement of parameters, such as the patient’s distance from the screen, reading speed, smallest readable letter size and reading acuity. Using this device Attia et al. recorded median binocular uncorrected and distance-corrected reading acuities of 0.18logMAR at intermediate distance (80 cm) and of 0.05logMAR and 0.01logMAR at near distance (40 cm) with AcrySof Restor, (Alcon, Laboratories, Ft. Worth, TX, USA) a diffractive bifocal IOL with + 3.0 D near addition [18]., For Lentis MPlus, (Oculentis GmbH, Berlin, Germany), a rotationally asymmetric bifocal IOL with a + 3.0 D near addition, Linz et al. found median monocular uncorrected and distance-corrected reading acuities of 0.30logMAR and 0.18logMAR for intermediate distance (80 cm) and of 0.18logMAR for near distance (40 cm) [19]. With the diffractive trifocal FineVision IOL, Attia et al. reported median binocular uncorrected and distance-corrected reading acuities of 0.10logMAR and 0.11logMAR for intermediate distance (80 cm) and of 0.11logMAR and 0.01logMAR for near distance (40 cm) [30]. We found Mini Well reading performance at intermediate distance is comparable to that of the FineVision IOL and better than that of bifocal IOLs. The same is true for uncorrected near reading acuity. With the trifocal IOL, on the other hand, patients achieved a better distance-corrected near reading acuity.

Photic phenomena are an intrinsic problem with all MIOLs since the diversion of light to different foci unavoidably causes superposition of multiple images on the retina [10, 31, 32]. All diffractive IOLs are known to cause Halos to some extent, especially Starbursts [14]. The Mini WELL should cause less dysphotopsia since it does not rely on diffractive optics. However, assessment of photic phenomena is difficult and highly depends on the inquiry technique. Mendicute et al. correctly observed that the outcome of these questionnaires may vary according to whether one asks open or closed questions [8]. Indeed, if patients were not at all specifically asked for photic phenomena, the amount reported would be much lower. It is therefore difficult to compare across different studies the significance of photic phenomena attributable to a given IOL. In inquiries that only used undirected questions (‘Do you experience any difficulties with your vision’) the incidence of dysphotopsia reported was low [9]. Explicitly asking for different types of photic phenomena yielded higher numbers [8]. Although several studies report high patient satisfaction and low incidence of halos and glare, dysphotopsia remains a major complaint among patients who are dissatisfied after MIOL implantation [33–35].

We used a computer-based simulator to assess perception of Halos and Glare, one which our group has experience of in studying trifocal IOLs [36]. We found the patients with the Mini WELL were mildly or not at all disturbed by Halos and Starbursts in daily life. However, we still regard this simulator as exploratory and so far, we restrained our analysis to being solely descriptive.

The clinical results reported herein should be considered in the light of some limitations, such as the limited number of patients enrolled or the lack of a control group. Future studies should also evaluate the Mini WELL IOL’s clinical performance with a longer follow-up period.

## Conclusions

Previous clinical and laboratory studies, which directly compared EDOF-IOLs and Trifocal lenses, concluded EDOF-IOLs provide good far and intermediate visual results but fall short of trifocal IOLs for near visual acuity at common reading distances [10, 13]. Our results suggest the Mini WELL is different, showing good near visual acuity in addition to very good intermediate distance visual acuity. Laboratory observations support this finding [21]. In general, the Mini WELL renders a level of visual rehabilitation similar to that of current trifocal IOLs. Overall perception of dysphotopsia is lower and less severe than in comparable IOLs. These results should be confirmed with increased patient numbers and longer follow-up periods.

## Data Availability

All generated or analyzed during this study are included in this published article.

## Abbreviations

CDVA: Corrected Distance Visual Acuity
CNVA: Corrected Near Visual Acuity
DCIVA: Distance-Corrected Intermediate Visual Acuity
DCNVA: Distance-Corrected Near Visual Acuity
EDOF: Extended Depth of Focus
ETDRS: Early Treatment Diabetic Retinopathy Study.
IOL: Intraocular Lens;
MIOLs: Multifocal Intraocular Lenses
UDVA: Uncorrected Distance Visual Acuity
UIVA: Uncorrected Intermediate Visual Acuity
UNVA: Uncorrected Near Visual Acuity

## References

[1] Auffarth GU, Rabsilber TM, Kohnen T, Holzer MP (2008). Design and optical principles of multifocal lenses. Ophthalmologe.

[2] Madrid-Costa D, Ruiz-Alcocer J, Ferrer-Blasco T, Garcia-Lazaro S, Montes-Mico R (2013). Optical quality differences between three multifocal intraocular lenses: bifocal low add, bifocal moderate add, and trifocal. J Refract Surg.

[3] Gatinel D, Pagnoulle C, Houbrechts Y, Gobin L (2011). Design and qualification of a diffractive trifocal optical profile for intraocular lenses. J Cataract Refract Surg.

[4] Bilbao-Calabuig R, Llovet-Rausell A, Ortega-Usobiaga J, Martinez-Del-Pozo M, Mayordomo-Cerda F, Segura-Albentosa C, Baviera J, Llovet-Osuna F. Visual outcomes following bilateral lmplantation of two diffractive trifocal IOLs in 10,084 eyes. Am J Ophthalmol. 2017;179:55-66. 10.1016/j.ajo.2017.04.013 28456547

[5] Alio JL, Montalban R, Pena-Garcia P, Soria FA, Vega-Estrada A (2013). Visual outcomes of a trifocal aspheric diffractive intraocular lens with microincision cataract surgery. J Refract Surg.

[6] Cochener B, Vryghem J, Rozot P, Lesieur G, Chevalier JP, Henry JM, David T, Lesueur L, Gatinel D, Ganem C, Blanckaert J, Van Acker E, Heireman S, Ghekiere S (2014). Clinical outcomes with a trifocal intraocular lens: a multicenter study. J Refract Surg.

[7] Lawless M, Hodge C, Reich J, Levitz L, Bhatt UK, McAlinden C, Roberts K, Roberts TV (2017). Visual and refractive outcomes following implantation of a new trifocal intraocular lens. Eye Vis (Lond).

[8] Mendicute J, Kapp A, Levy P, Krommes G, Arias-Puente A, Tomalla M, Barraquer E, Rozot P, Bouchut P (2016). Evaluation of visual outcomes and patient satisfaction after implantation of a diffractive trifocal intraocular lens. J Cataract Refract Surg.

[9] Cochener B, Concerto SG (2016). Clinical outcomes of a new extended range of vision intraocular lens: international multicenter Concerto study. J Cataract Refract Surg.

[10] Gatinel D, Loicq J (2016). Clinically relevant optical properties of bifocal, trifocal, and extended depth of focus intraocular lenses. J Refract Surg.

[11] Kaymak H, Hohn F, Breyer DR, Hagen P, Klabe K, Gerl RH, Mueller M, Auffarth GU, Gerl M, Kretz FT (2016). Functional results 3 months after implantation of an “extended range of vision” intraocular Lens. Klin Monatsbl Augenheilkd.

[12] Pedrotti E, Bruni E, Bonacci E, Badalamenti R, Mastropasqua R, Marchini G (2016). Comparative analysis of the clinical outcomes with a Monofocal and an extended range of vision intraocular Lens. J Refract Surg.

[13] Ruiz-Mesa Ramón, Abengózar-Vela Antonio, Aramburu Ana, Ruiz-Santos María (2017). Comparison of Visual Outcomes after Bilateral Implantation of Extended Range of Vision and Trifocal Intraocular Lenses. European Journal of Ophthalmology.

[14] de Silva SR, Evans JR, Kirthi V, Ziaei M, Leyland M (2016). Multifocal versus monofocal intraocular lenses after cataract extraction. Cochrane Database Syst Rev.

[15] de Vries NE, Nuijts RM (2013). Multifocal intraocular lenses in cataract surgery: literature review of benefits and side effects. J Cataract Refract Surg.

[16] Dexl AK, Schlogel H, Wolfbauer M, Grabner G (2010). Device for improving quantification of reading acuity and reading speed. J Refract Surg.

[17] Hirnschall N, Motaabbed JK, Dexl A, Grabner G, Findl O (2014). Evaluation of an electronic reading desk to measure reading acuity in pseudophakic patients. J Cataract Refract Surg.

[18] Attia MS, Khoramnia R, Auffarth GU, Kirchner M, Holzer MP (2016). Near and intermediate visual and reading performance of patients with a multifocal apodized diffractive intraocular lens using an electronic reading desk. J Cataract Refract Surg.

[19] Linz K, Attia MS, Khoramnia R, Tandogan T, Kretz FT, Auffarth GU (2016). Clinical evaluation of Reading performance using the Salzburg Reading desk with a refractive rotational asymmetric multifocal intraocular Lens. J Refract Surg.

[20] Savini G, Balducci N, Carbonara C, Rossi S, Altieri M, Frugis N, Zappulla E, Bellucci R, Alessio G (2019). Functional assessment of a new extended depth-of-focus intraocular lens. Eye (Lond).

[21] Dominguez-Vicent A, Esteve-Taboada JJ, Del Aguila-Carrasco AJ, Monsalvez-Romin D, Montes-Mico R (2016). In vitro optical quality comparison of 2 trifocal intraocular lenses and 1 progressive multifocal intraocular lens. J Cataract Refract Surg.

[22] Sheppard AL, Shah S, Bhatt U, Bhogal G, Wolffsohn JS (2013). Visual outcomes and subjective experience after bilateral implantation of a new diffractive trifocal intraocular lens. J Cataract Refract Surg.

[23] Jonker SM, Bauer NJ, Makhotkina NY, Berendschot TT, van den Biggelaar FJ (2015). Nuijts RM. Comparison of a trifocal intraocular lens with a +3.0 D bifocal IOL: results of a prospective randomized clinical trial. J Cataract Refract Surg.

[24] Mojzis P, Pena-Garcia P, Liehneova I, Ziak P, Alio JL (2014). Outcomes of a new diffractive trifocal intraocular lens. J Cataract Refract Surg.

[25] Kretz FT, Muller M, Gerl M, Gerl RH, Auffarth GU (2015). Binocular function to increase visual outcome in patients implanted with a diffractive trifocal intraocular lens. BMC Ophthalmol.

[26] Kretz FT, Breyer D, Diakonis VF, Klabe K, Henke F, Auffarth GU, Kaymak H (2015). Clinical outcomes after binocular implantation of a new trifocal diffractive intraocular Lens. J Ophthalmol.

[27] Alio JL, Grabner G, Plaza-Puche AB, Rasp M, Pinero DP, Seyeddain O, Rodriguez-Prats JL, Ayala MJ, Moreu R, Hohensinn M, Riha W, Dexl A (2011). Postoperative bilateral reading performance with 4 intraocular lens models: six-month results. J Cataract Refract Surg.

[28] Rasp M, Bachernegg A, Seyeddain O, Ruckhofer J, Emesz M, Stoiber J, Grabner G, Dexl AK (2012). Bilateral reading performance of 4 multifocal intraocular lens models and a monofocal intraocular lens under bright lighting conditions. J Cataract Refract Surg.

[29] Santhiago MR, Netto MV, Espindola RF, Mazurek MG, Gomes Bde A, Parede TR, Harooni H, Kara-Junior N (2010). Comparison of reading performance after bilateral implantation of multifocal intraocular lenses with +3.00 or +4.00 diopter addition. J Cataract Refract Surg.

[30] Attia MS, Auffarth GU, Khoramnia R, Linz K, Kretz FT (2015). Near and intermediate reading performance of a diffractive trifocal intraocular lens using a reading desk. J Cataract Refract Surg.

[31] Alba-Bueno F, Vega F, Millan MS (2014). Halos and multifocal intraocular lenses: origin and interpretation. Arch Soc Esp Oftalmol.

[32] Vega F, Alba-Bueno F, Millan MS, Varon C, Gil MA, Buil JA (2015). Halo and through-focus performance of four diffractive multifocal intraocular lenses. Invest Ophthalmol Vis Sci.

[33] de Vries NE, Webers CA, Touwslager WR, Bauer NJ, de Brabander J, Berendschot TT, Nuijts RM (2011). Dissatisfaction after implantation of multifocal intraocular lenses. J Cataract Refract Surg.

[34] Kamiya K, Hayashi K, Shimizu K, Negishi K, Sato M, Bissen-Miyajima H, Survey Working Group of the Japanese Society of C, Refractive S. Multifocal intraocular lens explantation: a case series of 50 eyes. Am J Ophthalmol 2014; 158: 215–220 e211. 10.1016/j.ajo.2014.04.010 24792105

[35] Davies EC, Pineda R (2016). Intraocular lens exchange surgery at a tertiary referral center: indications, complications, and visual outcomes. J Cataract Refract Surg.

[36] Kretz FT, Breyer D, Klabe K, Hagen P, Kaymak H, Koss MJ, Gerl M, Mueller M, Gerl RH, Auffarth GU (2015). Clinical outcomes after implantation of a trifocal Toric intraocular Lens. J Refract Surg.

